# Mitigation of cadmium-induced stress in maize via synergistic application of biochar and gibberellic acid to enhance morpho-physiological and biochemical traits

**DOI:** 10.1186/s12870-024-04805-2

**Published:** 2024-03-15

**Authors:** Tauseef Anwar, Huma Qureshi, Mah Jabeen, Wajid Zaman, Hayssam M. Ali

**Affiliations:** 1 Department of Botany, The Islamia University of Bahawalpur (Baghdad ul Jadeed Campus), Bahawalpur, 63100 Pakistan; 2 Department of Botany, University of Chakwal, Chakwal, 48800 Pakistan; 3 Department of Life Sciences, Yeungnam University, Gyeongsan, 38541 Republic of Korea; 4 Department of Botany and Microbiology, College of Science, King Saud University, Riyadh, 11451 Saudi Arabia

**Keywords:** Heavy metal stress, Soil remediation, Phytochemical response, Sustainable agriculture

## Abstract

Cadmium (Cd), being a heavy metal, tends to accumulate in soils primarily through industrial activities, agricultural practices, and atmospheric deposition. Maize, being a staple crop for many regions, is particularly vulnerable to Cd contamination, leading to compromised growth, reduced yields, and potential health risks for consumers. Biochar (BC), a carbon-rich material derived from the pyrolysis of organic matter has been shown to improve soil structure, nutrient retention and microbial activity. The choice of biochar as an ameliorative agent stems from its well-documented capacity to enhance soil quality and mitigate heavy metal stress. The study aims to contribute to the understanding of the efficacy of biochar in combination with GA_3_, a plant growth regulator known for its role in promoting various physiological processes, in mitigating the adverse effects of Cd stress. The detailed investigation into morpho-physiological attributes and biochemical responses under controlled laboratory conditions provides valuable insights into the potential benefits of these interventions. The experimental design consisted of three replicates in a complete randomized design (CRD), wherein soil, each containing 10 kg was subjected to varying concentrations of cadmium (0, 8 and 16 mg/kg) and biochar (0.75% w/w base). Twelve different treatment combinations were applied, involving the cultivation of 36 maize plants in soil contaminated with Cd (T1: Control (No Cd stress; T2: Mild Cd stress (8 mg Cd/kg soil); T3: Severe Cd stress (16 mg Cd/kg soil); T4: 10 ppm GA_3_ (No Cd stress); T5: 10 ppm GA_3_ + Mild Cd stress; T6: 10 ppm GA_3_ + Severe Cd stress; T7: 0.75% Biochar (No Cd stress); T8: 0.75% Biochar + Mild Cd stress; T9: 0.75% Biochar + Severe Cd stress; T10: 10 ppm GA_3_ + 0.75% Biochar (No Cd stress); T11: 10 ppm GA_3_ + 0.75% Biochar + Mild Cd stress; T12: 10 ppm GA3 + 0.75% Biochar + Severe Cd stress). The combined application of GA_3_ and BC significantly enhanced multiple parameters including germination (27.83%), root length (59.53%), shoot length (20.49%), leaf protein (121.53%), root protein (99.93%), shoot protein (33.65%), leaf phenolics (47.90%), root phenolics (25.82%), shoot phenolics (25.85%), leaf chlorophyll a (57.03%), leaf chlorophyll b (23.19%), total chlorophyll (43.77%), leaf malondialdehyde (125.07%), root malondialdehyde (78.03%) and shoot malondialdehyde (131.16%) across various Cd levels compared to the control group. The synergistic effect of GA_3_ and BC manifested in optimal leaf protein and malondialdehyde levels indicating induced tolerance and mitigation of Cd detrimental impact on plant growth. The enriched soils showed resistance to heavy metal toxicity emphasizing the potential of BC and GA_3_ as viable strategy for enhancing maize growth. The application of biochar and gibberellic acid emerges as an effective means to mitigate cadmium-induced stress in maize, presenting a promising avenue for sustainable agricultural practices.

## Introduction

The accumulation of elements such as lead, cadmium, mercury and others due to industrial activities, mining operations and inadequate waste management leads to heavy metal toxicity. This phenomenon poses significant threats to both ecosystems and human health [1]. The consequences encompass soil pollution, water contamination as well as severe health hazards like neurological disorders, organ impairment and potential carcinogenic effects [2]. In addition to other vital minerals, plants can easily take Cd from the soil through their roots. Like other heavy metals, Cd alters the structural makeup of plants and harms their morphological, physiological and biochemical processes resulting in a decline in agricultural yield. Plants exhibit a high sensitivity to cadmium leading to a significant impact on their growth and overall metabolism. [2]. Because Cd is unknown to play any part in the growth and development of crop plants, it is often not needed for crops. Therefore, even in low concentrations, Cd interferes with photosynthesis, alters the chloroplast’s ultrastructure boosts lipid peroxidation and increases the oxidative damage caused by reactive oxygen species.

One abiotic factor reducing agricultural productivity is soil heavy metal pollution. Additionally, heavy metals hinder photosynthesis and limit crop growth by competing with vital mineral nutrients and producing phytotoxicity. Cd accumulation in plants can result in a variety of physiological, metabolic and structural changes. Plant growth regulators are organic substances that can speed up, slow down or alter physiological processes in plants [10]. Low amounts of Cd are not detrimental to plants but higher levels of Cd chloride can impair root development as well as other elements of plant growth. Uncontaminated soils typically have a Cd content of less than 0.5 mg per kilogram, though this quantity might vary depending on the soil’s parent material. The rising levels of Cd significantly impede plant development and disrupt production processes. The type of plant, length of exposure, amount of absorption and sequestration in various parts are key determinants of anatomical anomalies. Much like other heavy metals, Cd induces oxidative damage in plants by triggering an overproduction of H_2_O_2_ and lipid peroxidation. The presence of reactive oxygen species (ROS) such as H_2_O_2_ and O^− 2^ scavenges antioxidant enzymes. [12]. Due to its greater suppleness and noxiousness, Cd is thought to be the most dangerous heavy metal, especially when it is found in agricultural regions [3]. Maize (*Zea mays*) is considered a significant contributor of dietary Cd for humans.

Maize is commonly used in the phyto-management of contaminated soils, especially soils contaminated with Cd and is also an effective AMF colonizer. Despite being exposed to Cd stress the maize plant produces a lot of biomass [4]. 

An essential signaling hormone for plants, gibberellic acid (GA_3_) activates a variety of physiological and developmental processes in plants such as root production, flowering, cell division, maturity and seed germination. GA_3_ also increases plants’ resistance to environmental challenges such as salt, cold, drought and heavy metal stress [5–7]. By controlling antioxidant enzyme activity, GA_3_ offers protection for plants against environmental challenges. Furthermore, it lowers the excessive levels of intracellular ROS produced when a situation is stressful. Exogenous GA_3_ has been shown to reduce oxidative stress in wheat caused by salt stress [14]. Furthermore, a study discovered that GA_3_ decreased Cd concentrations and reduced Cd stress while increasing sunflower yield. By enhancing antioxidant enzymes that mitigated the harmful effects, the GA_3_ treatment enhanced the development of pea plants under heavy metal stress [8–11]. GA_3_ under Cd stress speeds up soybean development, increases the plant’s chlorophyll content and increases the rate at which it assimilates CO_2_ [12, 13]. Gibberellins are a large class of naturally occurring compounds that regulate a multitude of plant growth processes including seed germination, stem lengthening and flowering. Gibberellic acid boosts plant resilience to abiotic stressors, improves seed germination and expands the size of fruits [14, 15]. Applying seed priming with plant growth regulators allows for the modification of plant phenotypes at any point in their life cycles from germination to senescence [16, 17]. 

Biochar (BC) is a carbon-rich material produced through pyrolysis, a process involving the heating of biomass in the absence of oxygen [18]. An effective method to reduce heavy metals (HMs) in soils is to utilize BC. Because of its greater capacity for adsorption and capacity to reduce HM concentrations in soils, BC has been used in HM-contaminated soils in previous years [19]. Given its fundamental nature, porous texture and energetic functional groups, its role in Cd immobilization is favorable. Soils that had been treated with BC displayed less Cd transport and buildup. By generating compounds and using cation exchange techniques in soils, BC can quickly adsorb Cd, Pb and Cu. The use of BC to remove Cd from soil has recently attracted a lot of interest. It has been shown that adding BC to soil improves its biological, chemical and physical characteristics and promotes plant development. Recycling discarded straws to produce valuable BC is both ecologically friendly and practical from an economic standpoint [20]. Furthermore, a great deal of study has shown how well wheat straw and maize straw BC work to immobilize heavy metals [21]. Recently, it was also suggested that using BC and hyperaccumulators together, which had a synergistic effect, could be used to clean up HM-contaminated soil [22]. BC could rapidly improve soil physicochemical characteristics to increase plant photosynthetic capacity and promote growth and increase the ability for hydration, nutrients and nutrition level retention [23]. 

While the existing literature has extensively explored the individual impacts of gibberellic acid and BC on plant responses to heavy metal stress, notably cadmium (Cd), a significant research gap lies in the investigation of their combined effects on maize growth under Cd stress. Understanding how these two interventions may interact and complement each other in mitigating Cd stress on maize could provide valuable insights for sustainable agricultural practices and soil remediation strategies. Therefore, the objective of the current study was to determine whether BC and gibberellic acid had any positive impacts on the growth of maize under Cd stress. The hypothesis suggests that the concurrent application of gibberellic acid and BC has the potential to mitigate the adverse impacts of Cd stress on maize. This amelioration is anticipated to manifest through enhancements in growth along with improvements in physiological and biochemical attributes.

## Materials and methodology

### Soil sampling and analysis

The soil samples were collected from Bahawalpur district (29.3954° N latitude and 71.6728° E longitude). Following collection, samples underwent a sequence of procedures to find trace metal ions [24]. Ten milliliters of nitric acid were added to an Erlenmeyer flask containing one gram of dried soil and the combination was then allowed to sit for night. Subsequently, flask underwent controlled heating at 200 °C, cooling and treatment with HNO_3_ and HClO_4_. Further heating at 280 °C followed until fumes from HClO_4_ became evident. Post-cooling, hydrochloric acid was introduced followed by another heating-cooling cycle. Whatman filter paper number 42 was used to filter the resulting solution after being mixed with 1% hydrochloric acid. Distinct soil attributes were evaluated using specific methods: pH determination was performed on soil-saturated paste with a pH meter, [25] soil organic content (SOC) was quantified using potassium dichromate method; [26] electrical conductivity (EC) was assessed by mixing soil and deionized water at a 1:10 ratio and subsequent analysis was conducted via EC meter; [27] soil phosphorus (P) analysis followed the Olsen method; [28] for soil potassium (K) analysis, ammonium acetate was employed to extract and flame photometry was used [29].

### Biochar’s (BC) composition and nutritional content

Biochar was generated using waste materials sourced from local fruits and vegetables (orange, potato) available at coordinates 30°11’29.8"N 71°28’48.8"E. The collected waste was dried under sunlight and then fragmented into small pieces. Following this, the material underwent pyrolysis at a temperature of 325 ± 5 °C [30, 31]. After completing the pyrolysis process the substance was cooled, crushed and ground into particles smaller than 2 mm. To prepare the BC, it was washed with distilled water to eliminate any impurities and any remaining ash residues [32]. Subsequently, the BC was air-dried in a well-ventilated space until it reached complete dryness. Finally, the deashed BC was properly stored for further use.

### Procurement and sterilization of seeds

FH-1036 hybrid seeds were procured from a local market of Bahawalpur city. For seed sterilization in the study, three rinses with ethanol (95%) followed by sodium hypochlorite (5%) were carried out. The process required 30 min of submersion of seeds in a sodium hypochlorite solution succeeded by three washes utilizing 95% ethanol.^34^

### Experimental setup

Powdered deashed BC was combined with 90% pure gibberellic acid 3 (GA_3_). The process began with the prepared deashed BC being ground into a fine, uniform powder. Then, using an analytical balance 10 ppm GA_3_ was accurately weighed and combined with the BC. In the end, this powder was quickly used as a soil addition after processing. A rate of 0.75% BC was manually added to the soil. Following the BC-soil mixture, pot filling was carried out. There were three quantities of cadmium (Cd) used: 0 Cd for the control group, 8 mg/kg and 16 mg/kg Cd for the treatment groups. To introduce Cd toxicity, cadmium nitrate tetrahydrate was employed. The salt’s specifications are as follows: brand: ALDRICH, CAS number: 10022-68-1, MDL number: MFCD00149626, product number: 642,045, batch number: MKCT3996. Cadmium nitrate was used to spike 35 kg of soil over 21 days to introduce toxicity from metal. The 35 kg of soil was put in a plastic container for the spiking technique and we carefully mixed in the cadmium nitrate solution while adding it gradually to ensure a uniform distribution. To avoid contamination the soil was covered after spiking and we gave it a 21-day incubation period. This incubation duration was chosen to enable cadmium and the soil matrix to interact over an extended period more closely mimicking real-world conditions.

### Treatment details

To ensure resilience each treatment was repeated three times. There were twelve distinct treatments in the study.

**T1:** Control (No Cd stress)

**T2:** Mild Cd stress (8 mg Cd/kg soil)

**T3:** Severe Cd stress (16 mg Cd/kg soil)

**T4:** 10 ppm GA_3_ (No Cd stress)

**T5:** 10 ppm GA_3_ + Mild Cd stress

**T6:** 10 ppm GA_3_ + Severe Cd stress

**T7:** 0.75% Biochar (No Cd stress)

**T8:** 0.75% Biochar + Mild Cd stress

**T9:** 0.75% Biochar + Severe Cd stress

**T10:** 10ppm GA_3_ + 0.75% Biochar (No Cd stress)

**T11:** 10ppm GA_3_ + 0.75% Biochar + Mild Cd stress

**T12:** 10ppm GA_3_ + 0.75% Biochar + Severe Cd stress

The experiment involved 36 maize plants cultivated in cadmium-contaminated soil, with three replicates in a complete randomized design.

### Fertilizer application

The application of recommended fertilizer rates for macronutrients involved 200 kg N, 150 kg P and 100 kg K per hectare [33]. Each test unit received proper fertilization with 100 g of phosphorus (P_2_O_5_), 50 g of potassium (K_2_O), and 150 g of nitrogen (N). The fertilizers used were diammonium phosphate ((NH_4_)_2_HPO_4_) for phosphorus, potassium sulfate (K_2_SO_4_) for potassium, and urea (CH_4_N_2_O) for nitrogen.

### Irrigation practices

Tap water was utilized to water the plants in these pots since irrigation was necessary for optimum productivity. Water was provided to the pot as necessary three to four days a week until the maize plants reached full physiological development. According to Danish and Zafar-ul-Hye, maize plants were irrigated to maintain 60% field capacity (FC) during a growth season [34]. The soil’s FC was determined using the soil-saturation paste method. The frequency of field capacity measurements was conducted once a week throughout the experimental period to monitor soil water retention levels.

### Crop harvest

Maize plants were harvested at the V10 Zadoks growth stage. Samples of roots, shoots and leaves were obtained to collect data.

### Measurement of root and shoot lengths

Each of the three plants in each group had its roots and shoots measured precisely in centimeters using a measuring tape.

### Measurement of protein contents

The total soluble protein was computed using the Afrasayab et al. method [35]. Frozen plant material was crushed in phosphate buffer (0.1 M pH 7.0) at a ratio of 1:4 (w/v). After 10 min of spinning the sample at 4 °C at 14,000 rpm, 2 ml of the Folin’s mixture was mixed with the supernatant (0.4 ml) and the sample was allowed to stand at room temperature for 15 min. Following that 0.2 ml of the phenol reagent created by Folin and Ciocalteu was mixed and allowed to sit at room temperature for 45 min to initiate the color development. Soluble protein concentration was calculated using a reference curve and the optical density at 750 nm.

### Measurement of phenolic contents

The measurement of soluble phenolics was done using the Folin-Ciocalteu method [36]. After the samples were incubated for an hour at 45 °C, a spectrophotometer (NanoDropTM spectrophotometer, USA) was used to detect the absorbance at 750 nm. The Folin-Ciocalteu reagent, Na_2_CO_3_ and 500 µl of redistilled water made up the blank. To determine the total phenolics a standard curve constructed with gallic acid was utilized. The results were reported as milligrams per gram of dry weight.

### Measurement of chlorophyll contents

Fresh leaves were homogenized in 80% acetone (v/v) and analyzed at wavelengths of 663, 645 and 480 nm using a spectrophotometer [37, 38]. Subsequently, the blend was subjected to filtration followed by centrifugation to remove insoluble particles. The resultant supernatant was carefully poured into a test tube.

$$Chlorophyll a \left(\frac{mg}{g}\right)=\frac{\left(12.7 \times A663\right)- \left(2.69 \times A645\right)\times V}{1000 \times W}$$

$$Chlorophyll b \left(\frac{mg}{g}\right)=\frac{\left(22.9 \times A645\right)- \left(4.68 \times A645\right)\times V}{1000 \times W}$$

$$Total \, Chlorophyll\, (mg/g) = Chlorophyll\, a + Chlorophyll\, b$$

### Measurement of malondialdehyde (MDA) contents

The approach was improved and used for the MDA determination [39]. The tissue material weighing 500 mg was ground in a mortar filled with sand and 5 ml of a 0.1 (w/v) trichloroacetic acid solution. Before dividing 1 ml of the supernatant into two Eppendorf tubes with a concentration of 0.5 ml each, the homogenate was centrifuged at 10,000 x g for 20 min. The mixture was then incubated at 96 °C for 30 min after the aliquots were combined with 1 ml of a 0.5% (w/v) thiobarbituric acid in 20% thiobarbituric acid solution. By immersing reaction tubes in an ice bath, reaction was stopped after five minutes of 10,000 x g centrifugation and the absorbance of the supernatant was measured at 532 nm. The non-specific absorption value was eliminated at 600 nm. The amount of MDA-TBA complex was calculated using an extinction coefficient of 155 mM^− 1^cm^− 1^.

### Statistical analysis

The collected data underwent standard statistical analysis using OriginPro 2021 software. To determine the significance of differences between treatment groups, standard one-way analysis of variance (ANOVA) and Fisher’s LSD test were employed [40, 41]. 

## Results

The initial soil sample exhibited a pH of 8.26, electrical conductivity (EC) of 3.11 dS/m^− 1^, 0.65% organic matter content, 0.04 g/kg total nitrogen (N), 9.87 mg/kg extractable phosphorus (EP), and 139 mg/kg available potassium (AK) concentration. The properties of the biochar created during the first experiment phase, both physically and chemically are outlined in Table 1.

**Table 1:** Soil, biochar and irrigation attributes before the commencement of the experiment

Soil	Values	Biochar	Values	Irrigation	Values
pH	8.26	pH	8.21	pH	6.94
EC*e* (dS/m)	3.11	EC*e* (dS/m)	3.05	EC (µS/cm)	471
SOC (%)	0.65	Volatile Matter (%)	25	Carbonates (meq. /L)	0.00
TN (%)	0.04	Fixed carbon (%)	45	bicarbonates (meq./L)	4.11
EP (mg/kg)	9.87	TN (%)	0.07	Chloride (meq. /L)	0.10
AK (mg/kg)	139	TP (%)	0.19	Ca + Mg (meq. /L)	2.99
Sand (%)	25	TK (%)	0.33	Sodium (mg/L)	123
Silt (%)	40	TCd (µg/g)	0.09	---	---
Clay (%)	35	Particle Size	< 2 mm	---	---
Texture	Clay Loam		---	---	---

TN = Total Nitrogen; EP = Extractable Phosphorus; AK = Available Potassium; CEC = Cation Exchange Capacity; EC = Electrical Conductivity; TCd = Total Cadmium

### Effect of GA_3_ and biochar on maize germination percentage, root and shoot lengths under Cd stress

**Fig. 1:**
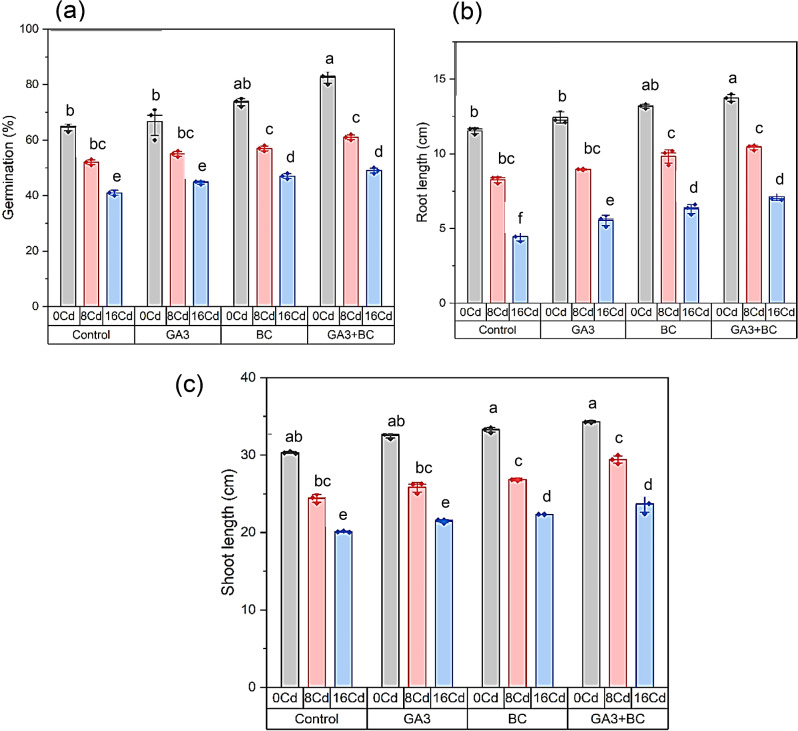
Influence of toxic metal on the efficacy of the biochar-GA3 intervention for enhancing Cadmium tolerance in maize growth parameters: (**a**): germination percentage (%), (**b**): root length (cm), (**c**): shoot length (cm). (GA_3_ = Gibberellic acid; BC = Biochar)

In the absence of Cd stress (0 Cd), the germination rate exhibited an increase to 64.66%. However, in the 8 Cd treatment involving Cd stress, the germination rate decreased to 52%. Under high Cd stress conditions (16 Cd), the germination rate was further reduced to 41%. Conversely, applying only GA_3_ treatment without any stress (0 Cd) resulted in an elevated germination rate of 66.66%, signifying a noteworthy 3.09% increase over the 0 Cd control. Similarly, the combined application of GA_3_ treatment and 8 Cd stress yielded a germination rate of 55%, representing a substantial 5.76% increase compared to the 8 Cd control. Compared to the 16 Cd control, the germination rate increased significantly by 8.94–44.66% when exposed to high Cd stress (16 Cd) conditions in conjunction with GA_3_ treatment. In the absence of stress (0 Cd) but with BC treatment, the germination rate significantly increased to 73.66%, marking a remarkable 13.91% increase over the 0 Cd control. Under BC treatment and 8 Cd stress, the germination rate was recorded at 57%, showcasing a substantial 9.61% increase compared to the 8 Cd control. Under conditions of high Cd stress (16 Cd) with BC treatment, the germination rate ascended to 47%, reflecting a notable 14.63% increase compared to the 16 Cd control. With GA_3_ + BC treatment in the absence of Cd stress (0 Cd), the germination rate surged to 82.66%, representing a substantial 27.83% increase over the 0 Cd control. Exposure to GA_3_ + BC treatment and 8 Cd stress resulted in a germination rate of 61%, marking a significant 17.30% increase compared to the 8 Cd control. Under high Cd stress (16 Cd) conditions with GA_3_ + BC treatment, the germination rate reached 49%, showing a noteworthy 19.51% increase compared to the 16 Cd control (Fig. 1a).

Compared to the control group, exposure to 0 Cd stress resulted in an 11.54% increase in root length. However, in the 8 Cd stress treatment, the root length declined to 8.27%. Moreover, under high Cd-stress conditions (16 Cd), the root length experienced a further reduction to 4.47%. In contrast, administering only GA_3_ treatment without stress (0 Cd) led to an elevation in root length to 12.42%, signifying a significant 7.65% increase over the 0 Cd control. Similarly, the root length increased to 8.95% after GA_3_ treatment and 8 Cd stress was applied, which is a significant 8.31% increase over the 8 Cd control. Compared to the 16 Cd control, the root length measured 5.54% when exposed to high Cd stress (16 Cd) combined with GA_3_ treatment. This represents a significant 23.99% increase. Likewise, under the absence of stress (0 Cd) but with BC treatment, the root length saw a noteworthy rise to 13.18%, marking a remarkable 14.21% increase over the 0 Cd control. Exposure to BC treatment and 8 Cd stress resulted in a root length of 9.82%, signifying a substantial 18.83% increase compared to the 8 Cd control. When exposed to a high concentration of Cd (16 Cd) through BC treatment, the root length increased to 6.30%, showing a noteworthy 40.99% increase over the 16 Cd control. When GA_3_ + BC was applied and there was no Cd stress, the root length increased to 13.73%, which is a significant 18.99% increase over the 0 Cd control. After the GA_3_ + BC treatment and 8 Cd stress, the root length was 10.42%, which is a significant 26.07% increase over the 8 Cd control. With GA_3_ + BC treatment, the root length reached 7.13% under high Cd stress (16 Cd) conditions, indicating a significant 59.53% increase over the 16 Cd control (Fig. 1b).

Compared to the control, 0 Cd stress increased shoot length by 30.31%. Under 8 Cd stress, it decreased to 24.41%, and further to 20.09% under 16 Cd stress. GA_3_ treatment in 0 Cd conditions raised shoot length to 32.53%, a significant 3.09% increase. Similarly, GA_3_ treatment with 8 Cd stress yielded 25.84% shoot length, a substantial 5.76% increase over 8 Cd control. High Cd stress (16 Cd) with GA_3_ treatment resulted in 21.47% shoot length, a significant 8.92% increase over 16 Cd control. BC treatment in the absence of stress (0 Cd) significantly boosted shoot length to 33.24%, a remarkable 9.63% increase. BC treatment with 8 Cd stress led to 26.85% shoot length, a substantial 10.01% increase over 8 Cd control. Under high Cd stress (16 Cd) with BC treatment, shoot length reached 22.33%, showing a significant 11.11% increase over 16 Cd control. GA_3_ + BC treatment and 0 Cd stress resulted in 34.30% shoot length, a substantial 13.13% increase. With GA_3_ + BC treatment and 8 Cd stress, shoot length was 29.41%, representing a substantial 20.49% increase over 8 Cd control. Under high Cd stress (16 Cd) with GA_3_ + BC treatment, shoot length reached 23.66%, showing a significant 17.72% increase over 16 Cd control (Fig. 1c).

### Effect of GA_3_ and biochar on maize protein contents of root, shoot, and leaves under Cd stress

**Fig. 2:**
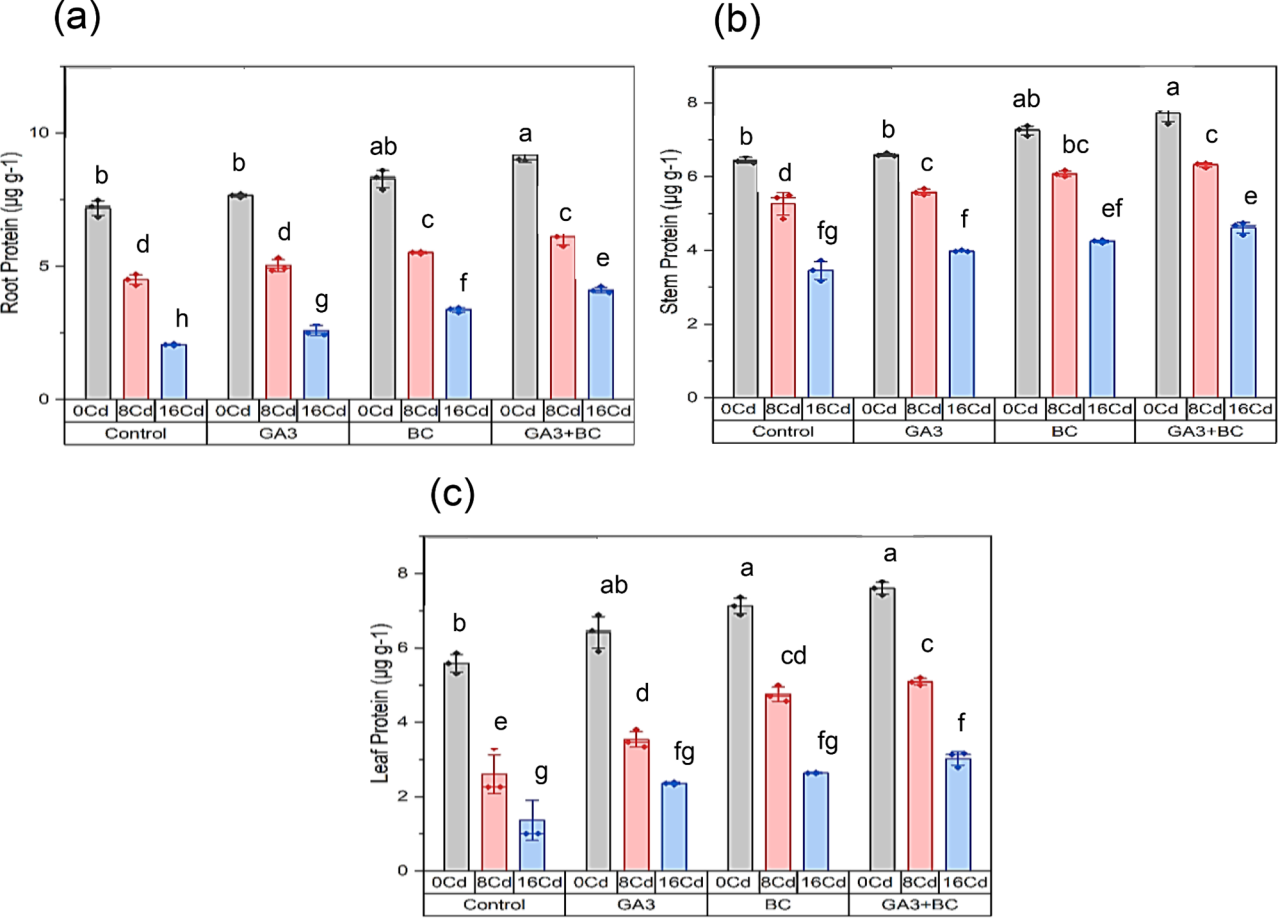
Influence of toxic metal on the efficacy of the biochar-GA_3_ intervention for enhancing Cadmium tolerance in maize growth parameters: (**a**): root protein (µg g^-1^**b**): shoot protein (µg g^-1^**c**): leaf protein (µg g^-1^_3_ = Gibberellic acid; BC = Biochar)

In comparison to the control, exposure to 0 Cd stress resulted in a 7.19% increase in root protein. However, under 8 Cd stress, it decreased to 4.49%, and further to 2.05% under 16 Cd stress. The application of GA_3_ treatment in 0 Cd conditions elevated root protein to 7.65%, signifying a significant 6.48% increase. Similarly, GA_3_ treatment with 8 Cd stress yielded 5.02% root protein, a substantial 11.77% increase over the 8 Cd control. High Cd stress (16 Cd) with GA_3_ treatment resulted in 2.59% root protein, indicating a significant 25.84% increase over the 16 Cd control. In the absence of stress (0 Cd) but with BC treatment, root protein significantly increased to 8.27%, marking a remarkable 15.09% increase. BC treatment with 8 Cd stress led to 5.51% root protein, representing a substantial 22.57% increase over the 8 Cd control. Under high Cd stress (16 Cd) with BC treatment, root protein reached 3.36%, showing a significant 63.54% increase over the 16 Cd control. GA_3_ + BC treatment and 0 Cd stress resulted in 9.18% root protein, representing a substantial 27.76% increase. With GA_3_ + BC treatment and 8 Cd stress, root protein was 6.11%, showcasing a substantial 36.00% increase over the 8 Cd control. Under high Cd stress (16 Cd) with GA_3_ + BC treatment, root protein reached 4.11%, indicating a significant 99.93% increase over the 16 Cd control (Fig. 2a).

In comparison to the control, exposure to 0 Cd stress increased leaf protein by 5.58%. However, under 8 Cd stress, it decreased to 2.60%, and further to 1.36% under 16 Cd stress. GA_3_ treatment in 0 Cd conditions raised leaf protein to 6.41%, a significant 14.92% increase. Similarly, GA_3_ treatment with 8 Cd stress yielded 3.53% leaf protein, representing a substantial 35.77% increase over the 8 Cd control. High Cd stress (16 Cd) with GA_3_ treatment resulted in 2.35% leaf protein, a significant 72.52% increase over the 16 Cd control. In the absence of stress (0 Cd) but with BC treatment, leaf protein significantly increased to 7.13%, marking a remarkable 27.7% increase. BC treatment with 8 Cd stress led to 4.75% leaf protein, a substantial 82.53% increase over the 8 Cd control. Under high Cd stress (16 Cd) with BC treatment, leaf protein reached 2.63%, showing a significant 92.96% increase over the 16 Cd control. GA_3_ + BC treatment and 0 Cd stress resulted in 7.60% leaf protein, representing a substantial 36.19% increase. With GA_3_ + BC treatment and 8 Cd stress, leaf protein was 5.09%, showcasing a substantial 95.54% increase over the 8 Cd control. Under high Cd stress (16 Cd) with GA_3_ + BC treatment, leaf protein reached 3.02%, indicating a significant 121.53% increase over the 16 Cd control (Fig. 2c).

### Effect of GA_3_ and biochar on maize phenolic contents of root, shoot, and leaves under Cd stress

**Fig. 3:**
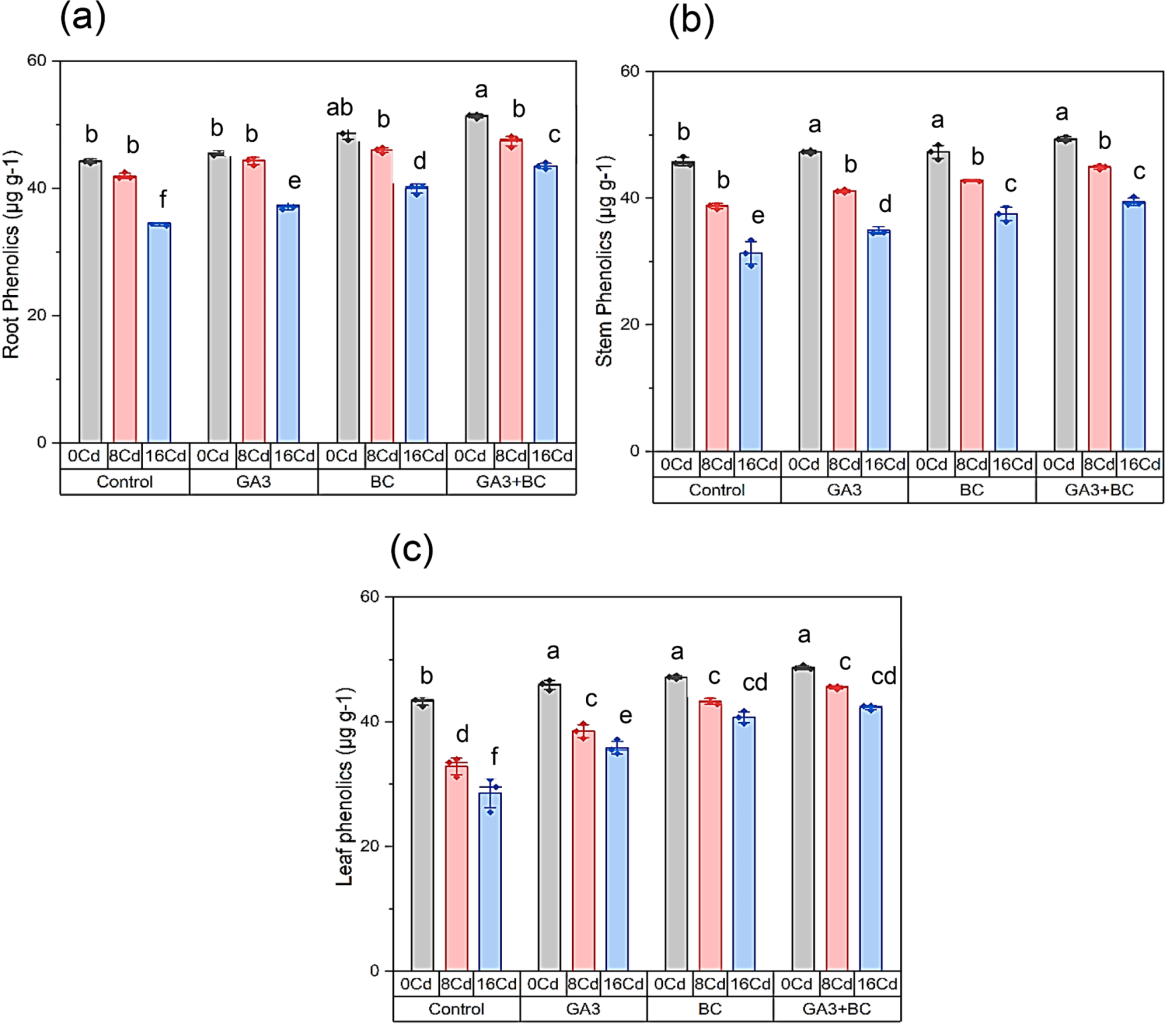
Influence of toxic metal on the efficacy of the biochar-GA_3_ intervention for enhancing Cadmium tolerance in maize growth parameters: (**a**): root phenolics (µg g^− 1^), (**b**): shoot phenolics (µg g^− 1^), (**c**): leaf phenolics (µg g^− 1^). (GA_3_ = Gibberellic acid; BC = Biochar)

Relative to the control, exposure to 0 Cd stress resulted in a 44.28% increase in root phenolic content. However, under 8 Cd stress, it decreased to 41.92%, and further to 34.56% under 16 Cd stress. The application of GA_3_ treatment in 0 Cd conditions elevated root phenolic content to 45.56%, signifying a significant 2.89% increase. Similarly, GA_3_ treatment with 8 Cd stress yielded 44.28% root phenolic content, representing a substantial 5.63% increase over the 8 Cd control. High Cd stress (16 Cd) with GA_3_ treatment resulted in 37.34% root phenolic content, indicating a significant 8.02% increase over the 16 Cd control. In the absence of stress (0 Cd) but with BC treatment, root phenolic content significantly increased to 48.66%, marking a remarkable 9.90% increase. BC treatment with 8 Cd stress led to 45.99% root phenolic content, representing a substantial 9.69% increase over the 8 Cd control. Under high Cd stress (16 Cd) with BC treatment, root phenolic content reached 39.98%, showing a significant 15.66% increase over the 16 Cd control. GA_3_ + BC treatment and 0 Cd stress resulted in 51.32% root phenolic content, representing a substantial 15.90% increase. With GA_3_ + BC treatment and 8 Cd stress, root phenolic content was 47.39%, showcasing a substantial 13.04% increase over the 8 Cd control. Under high Cd stress (16 Cd) with GA_3_ + BC treatment, root phenolic content reached 43.49%, indicating a significant 25.82% increase over the 16 Cd control (Fig. 3a).

Compared to the control, exposure to 0 Cd stress increased shoot phenolic content by 45.79%. However, under 8 Cd stress, it decreased to 38.75%, and further to 31.35% under 16 Cd stress. GA_3_ treatment in 0 Cd conditions raised shoot phenolic content to 47.31%, a significant 3.31% increase. Similarly, GA_3_ treatment with 8 Cd stress yielded 41.11% shoot phenolic content, representing a substantial 6.08% increase over the 8 Cd control. High Cd stress (16 Cd) with GA_3_ treatment resulted in 34.94% shoot phenolic content, indicating a significant 11.46% increase over the 16 Cd control. In the absence of stress (0 Cd) but with BC treatment, shoot phenolic content significantly increased to 47.29%, marking a remarkable 3.27% increase. BC treatment with 8 Cd stress led to 42.88% shoot phenolic content, representing a substantial 10.63% increase over the 8 Cd control. Under high Cd stress (16 Cd) with BC treatment, shoot phenolic content reached 37.51%, showing a significant 19.66% increase over the 16 Cd control. GA_3_ + BC treatment and 0 Cd stress resulted in 49.36% shoot phenolic content, representing a substantial 7.78% increase. With GA_3_ + BC treatment and 8 Cd stress, shoot phenolic content was 44.90%, showcasing a substantial 15.86% increase over the 8 Cd control. Under high Cd stress (16 Cd) with GA_3_ + BC treatment, shoot phenolic content reached 39.45%, indicating a significant 25.85% increase over the 16 Cd control (Fig. 3b).

Compared to the control, exposure to 0 Cd stress increased leaf phenolic content by 43.32%. However, under 8 Cd stress, it decreased to 32.85%, and further to 28.59% under 16 Cd stress. GA_3_ treatment in 0 Cd conditions raised leaf phenolic content to 45.90%, a significant 5.95% increase. Similarly, GA_3_ treatment with 8 Cd stress yielded 38.50% leaf phenolic content, representing a substantial 17.17% increase over the 8 Cd control. High Cd stress (16 Cd) with GA_3_ treatment resulted in 35.84% leaf phenolic content, indicating a significant 25.35% increase over the 16 Cd control. In the absence of stress (0 Cd) but with BC treatment, leaf phenolic content significantly increased to 47.14%, marking a remarkable 8.80% increase. BC treatment with 8 Cd stress led to 4.75% leaf phenolic content, representing a substantial 82.53% increase over the 8 Cd control. Under high Cd stress (16 Cd) with BC treatment, leaf phenolic content reached 40.71%, showing a significant 42.38% increase over the 16 Cd control. GA_3_ + BC treatment and 0 Cd stress resulted in 48.71% leaf phenolic content, representing a substantial 12.43% increase. With GA_3_ + BC treatment and 8 Cd stress, leaf phenolic content was 45.49%, showcasing a substantial 38.45% increase over the 8 Cd control. Under high Cd stress (16 Cd) with GA_3_ + BC treatment, leaf phenolic content reached 42.29%, indicating a significant 47.90% increase compared to the control (16 Cd) (Fig. 3c).

### Effect of GA_3_ and biochar on maize leaf chlorophyll a, b and total chlorophyll contents under Cd stress

**Fig. 4:**
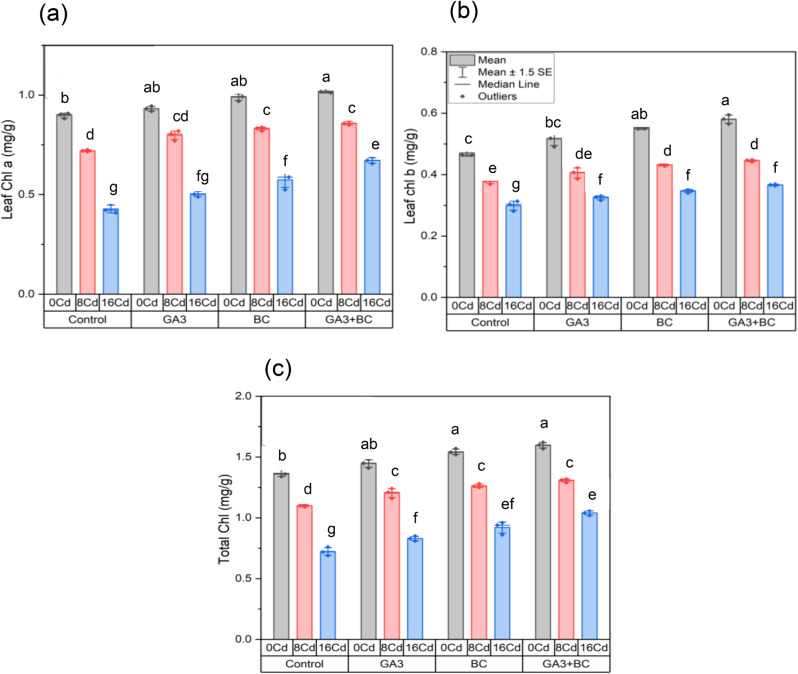
Influence of toxic metal on the efficacy of the biochar-GA_3_ intervention for enhancing Cadmium tolerance in maize growth parameters: (**a**): leaf chlorophyll a (mg/g), (**b**): leaf chlorophyll b (mg/g), (**c**): total chlorophyll (mg/g). (GA_3_ = Gibberellic acid; BC = Biochar)

Compared to the control, 0 Cd stress increased leaf chlorophyll a by 0.89%. Under 8 Cd stress, it decreased to 0.72%, and further to 0.42% under 16 Cd stress. Applying only GA_3_ treatment in 0 Cd conditions raised leaf chlorophyll a to 0.93%, indicating a significant 3.79% increase. Similarly, GA_3_ treatment with 8 Cd stress resulted in 0.79% leaf chlorophyll a, representing a substantial 10.81% increase over the 8 Cd control. Leaf chlorophyll a concentration increased significantly by 17.71–0.50% under high Cd stress (16 Cd) with GA_3_ treatment. BC treatment in the absence of stress (0 Cd) significantly boosted leaf chlorophyll a to 0.98%, indicating a remarkable 10.31% increase. BC treatment with 8 Cd stress resulted in 0.83% leaf chlorophyll a, representing a substantial 15.27% increase over the 8 Cd control. Under high Cd stress (16 Cd) with BC treatment, leaf chlorophyll a reached 0.57%, showing a significant 34.17% increase over the 16 Cd control. GA_3_ + BC treatment and 0 Cd stress led to 1.01% leaf chlorophyll a, representing a substantial 13.64% increase. When subjected to the GA_3_ + BC treatment and 8 Cd stress, leaf chlorophyll a was 0.85%, signifying a substantial 19.28% increase over the 8 Cd control. With GA_3_ + BC treatment, leaf chlorophyll a reached 0.67% under high Cd stress (16 Cd), indicating a significant 57.03% increase over the control (16 Cd) (Fig. 4a).

Relative to the control, 0 Cd stress induced a 0.47% increase in leaf chlorophyll b. In 8 Cd stress treatment, leaf chlorophyll b decreased to 0.37%, and further to 0.29% under 16 Cd stress. Applying only GA_3_ treatment in 0 Cd conditions led to an increase in leaf chlorophyll b to 0.51%, signifying a significant 9.69% rise. Similarly, GA_3_ treatment with 8 Cd stress yielded a leaf chlorophyll b of 0.40%, representing a substantial 7.32% increase over the 8 Cd control. High Cd stress (16 Cd) with GA_3_ treatment resulted in leaf chlorophyll b of 0.32%, indicating a significant 8.56% increase over the 16 Cd control. BC treatment without stress (0 Cd) significantly elevated leaf chlorophyll b to 0.55%, indicating a remarkable 17.66% increase. BC treatment with 8 Cd stress resulted in leaf chlorophyll b of 0.43%, representing a substantial 14.23% increase over the 8 Cd control. Under high Cd stress (16 Cd) conditions with BC treatment, leaf chlorophyll b reached 0.34%, showing a significant 15.81% increase compared to the control (16 Cd). GA_3_ + BC treatment and 0 Cd stress led to leaf chlorophyll b of 0.58%, representing a substantial 23.19% increase. When subjected to the GA_3_ + BC treatment and 8 Cd stress, leaf chlorophyll b was 0.44%, signifying a substantial 17.87% increase over the 8 Cd control. With GA_3_ + BC treatment, leaf chlorophyll b reached 0.36% under high Cd stress (16 Cd), indicating a significant 22.53% increase over the control (16 Cd) (Fig. 4b).

The total chlorophyll increased by 1.36% under 0 Cd stress compared to the control. Total chlorophyll dropped to 1.1% under the 8 Cd stress treatment and then to 0.72% under the 16 Cd stress. Applying only GA_3_ treatment in 0 Cd conditions increased total chlorophyll to 1.44%, signifying a significant 5.85% rise. Similarly, GA_3_ treatment with 8 Cd stress yielded a total chlorophyll of 1.20%, representing a substantial 9.39% increase over the 8 Cd control. Total chlorophyll was 0.83% after GA_3_ treatment of a high Cd stress (16 Cd), a significant 14.74% increase over the 16 Cd control. BC treatment without stress (0 Cd) significantly elevated total chlorophyll to 1.54%, indicating a remarkable 12.92% increase. BC treatment with 8 Cd stress resulted in a total chlorophyll of 1.26%, representing a substantial 14.84% increase over the 8 Cd control. Under high Cd stress (16 Cd) conditions with BC treatment, total chlorophyll reached 0.92%, showing a significant 27.18% increase compared to the control (16 Cd). GA_3_ + BC treatment and 0 Cd stress led to total chlorophyll of 1.59%, representing a substantial 16.82% increase. When subjected to the GA_3_ + BC treatment and 8 Cd stress, total chlorophyll was 1.30%, signifying a substantial 18.78% increase over the 8 Cd control. Under high Cd stress (16 Cd) conditions with GA_3_ + BC treatment, total chlorophyll reached 1.04%, showing a significant 43.77% increase compared to the control (16 Cd) (Fig. 4c).

### Effect of GA_3_ and biochar on maize malondialdehyde (MDA) contents of root, shoot, and leaves under Cd stress

**Fig. 5:**
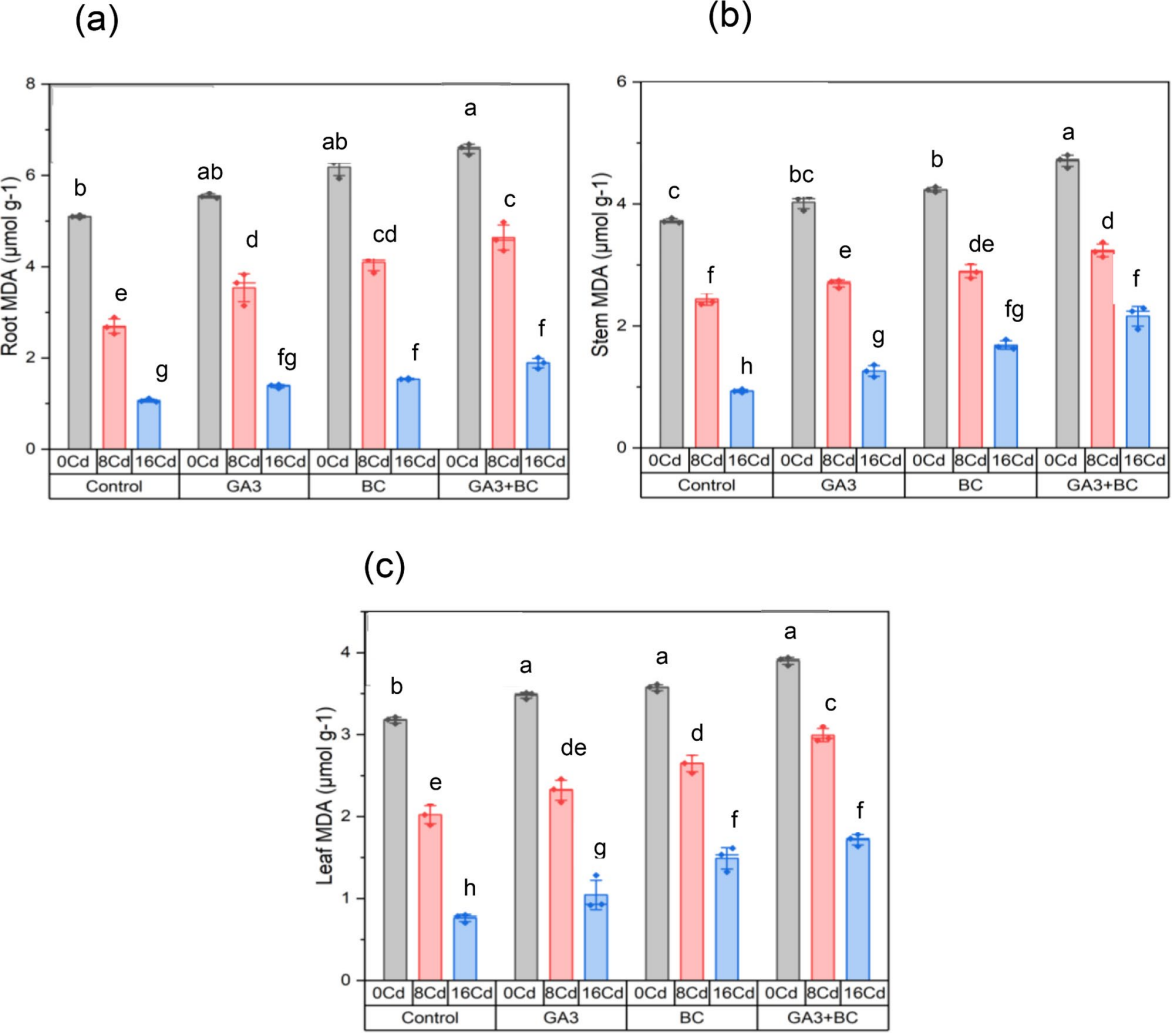
Influence of toxic metal on the efficacy of the biochar-GA_3_ intervention for enhancing Cadmium tolerance in maize growth parameters: (**a**): root MDA (µmol g^-1^**b**): shoot MDA (µmol g^-1^**c**): leaf MDA (µmol g^-1^_3_ = Gibberellic acid; BC = Biochar)

Compared to the control group, 0 Cd stress led to a 5.09% increase in root MDA levels. Under 8 Cd stress treatment, root MDA decreased to 2.69%, further diminishing to 1.06% under 16 Cd stress. Applying only GA_3_ treatment in 0 Cd conditions elevated root MDA to 5.55%, signifying a significant 8.88% rise. Similarly, GA_3_ treatment with 8 Cd stress resulted in root MDA of 3.53%, representing a substantial 31.23% increase over the 8 Cd control. High Cd stress (16 Cd) with GA_3_ treatment led to root MDA of 1.38%, indicating a significant 30.40% increase compared to the 16 Cd control. BC treatment without stress (0 Cd) significantly elevated root MDA to 6.18%, indicating a remarkable 21.24% increase. BC treatment and 8 Cd stress resulted in root MDA of 4.09%, representing a substantial 51.98% increase over the 8 Cd control. Under high Cd stress (16 Cd) conditions with BC treatment, root MDA reached 1.53%, showing a significant 45.02% increase compared to the 16 Cd control. GA_3_ + BC treatment and 0 Cd stress led to root MDA of 6.58%, representing a substantial 29.07% increase. When subjected to the GA_3_ + BC treatment and 8 Cd stress, root MDA was 4.63%, signifying a substantial 71.97% increase over the 8 Cd control. Root MDA under high Cd stress (16 Cd) conditions with GA_3_ + BC treatment was 1.88%, indicating a significant increase of 78.03% over the 16 Cd control (Fig. 5a).

Compared to the control group, 0 Cd stress led to a 3.72% increase in shoot MDA levels. Under 8 Cd stress treatment, shoot MDA decreased to 2.44%, further diminishing to 0.93% under 16 Cd stress. Applying only GA_3_ treatment in 0 Cd conditions elevated shoot MDA to 4.02%, signifying a significant 8.16% rise. Similarly, GA_3_ treatment with 8 Cd stress resulted in shoot MDA of 2.69%, representing a substantial 10.43% increase over the 8 Cd control. With GA_3_ treatment, high Cd stress (16 Cd) resulted in a shoot MDA of 1.26%, a significant 35.11% increase over the 16 Cd control. BC treatment without stress (0 Cd) significantly elevated shoot MDA to 4.23%, indicating a remarkable 13.70% increase. BC treatment and 8 Cd stress resulted in a shoot MDA of 2.89%, representing a substantial 18.73% increase over the 8 Cd control. Under high Cd stress (16 Cd) conditions with BC treatment, shoot MDA reached 1.68%, showing a significant 80.51% increase compared to the 16 Cd control. GA_3_ + BC treatment and 0 Cd stress led to shooting MDA of 4.70%, representing a substantial 26.36% increase. When subjected to the GA_3_ + BC treatment and 8 Cd stress, shoot MDA was 3.23%, signifying a substantial 32.70% increase over the 8 Cd control. Shoot MDA reached 2.15% under high Cd stress (16 Cd) conditions with GA_3_ + BC treatment, indicating a significant 131.16% increase compared to the 16 Cd control (Fig. 5b).

Exposure to 0 Cd stress increased leaf MDA levels by 3.17% when compared to the control group. Under the 8 Cd stress treatment, leaf MDA levels decreased to 2.02%, and under high Cd-stress conditions (16 Cd), they further declined to 0.76%. In contrast, when solely applying GA_3_ treatment without any stress (0 Cd), leaf MDA levels increased to 3.47%, signifying a significant 9.52% increase over the 0 Cd control. Similarly, with the application of GA_3_ treatment and 8 Cd stress, leaf MDA levels reached 2.32%, representing a substantial 14.71% increase compared to the 8 Cd control. With GA_3_ treatment, the leaf MDA levels under high Cd stress (16 Cd) were 1.04%, indicating a significant 36.91% increase over the 16 Cd control. Likewise, with the BC treatment in the absence of stress (0 Cd), leaf MDA levels significantly rose to 3.57%, indicating a remarkable 12.45% increase over the 0 Cd control. When subjected to BC treatment and 8 Cd stress, leaf MDA levels were 2.64%, representing a substantial 30.82% increase as compared to the 8 Cd control. Leaf MDA levels under high Cd stress (16 Cd) conditions with BC treatment reached 1.49%, indicating a significant 95.42% increase over the 16 Cd control. Leaf MDA levels in the GA_3_ + BC treatment and 0 Cd stress condition increased to 3.90%, which is a significant 22.76% increase over the 0 Cd control. In comparison to the 8 Cd control, leaf MDA levels were 2.99% after being exposed to the GA_3_ + BC treatment and 8 Cd stress, a significant 47.96% increase. Leaf MDA levels with GA_3_ + BC treatment reached 1.71% under high Cd stress (16 Cd), indicating a significant 125.07% increase over the 16 Cd control (Fig. 5c).

## Discussion

The presence of Cd was found to impact the percentage of germination significantly. Cd was found to reduce seed germination in four wheat cultivars, varying based on dosage and specific cultivar, mirroring findings from earlier [40–42]. According to French and Iyer-Pascuzzi, biochar accelerates germination and shortens the time it takes for two tomato genotypes to sprout [43]. According to Groot and Karssen, gibberellic acid regulates seed germination [44]. These findings align with the previous study which administered GA_3_ to barley plants that were under salt stress and noted decreased Zn, Fe, Co, Pb, Cr, Cd, and Mn ion accumulation [45]. Plants often decrease root length and surface area to minimize exposure to soil metal toxicity [46]. Heavy metal stress has been associated with reduced shoot and root lengths, along with diminished fresh and dry biomass in sunflowers. This reflects the results observed in our study [47]. The GA_3_ treatment led to improved shoot length compared to the control group, especially at higher Cd levels. Similar research demonstrates that adding GA (50 M) to *Brassica napus* grown in Cd (0, 50 and 100 M) hydroponics can increase plant height compared to utilizing solely Cd hydroponics, which lends credence to our findings [48]. The observed effect may be attributed to the roles of gibberellins in protein synthesis metabolism, cell division and growth, with significant changes in shoot length resulting from the application of biochar [49, 50].

The protein pool significantly decreased after Cd treatments. The overall protein concentration of spring maize was considerably (*p* ≤ 0.01) decreased by Cu and Ni stress [51]. The application of GA_3_ considerably slowed down the growth of maize plants as well as the detrimental effects of drought stress. Plants subjected to BC plus *T. asperellum* plus Cu showed higher levels of leaf protein compared to spring maize treated with Cu alone [52]. Past research indicates that incorporating GA_3_ into a *C. vulgaris* culture exposed to heavy metals increased the amount of protein. When BC is administered, antioxidant levels rise. This helps to defer or stop the oxidation of plant cells, proteins, lipids, and other vital components by stopping the start or spread of oxidative chain reactions [53]. 

Our findings support earlier research by showing a detrimental effect of Cd on protein accumulation it has also been seen in chamomile plants that Cd has a detrimental effect on soluble protein accumulation, which suggests that proteins are actively being metabolized and may be oxidized [54]. Contrary to the current study, it was previously discovered that Cd enhanced total phenolics in the *Lepidium sativum* when CdCl_2_ was given at low dosages (0.5 mg L^− 1^), but that it decreased in large doses [55]. The application of GA_3_ leads to higher leaf protein content compared to the control group at all Cd concentrations. These findings are supported by the studies, Rhizobium inoculation resulted in an uptick in protein levels, however, the use of GA_3_ improved this reaction even more [56]. Under Cd contamination in Biochar and Ascorbic Acid, total soluble protein levels dramatically rose but were not different from the control group [57]. When treatment in combination with Fe-BC was compared to Cd stress alone, the total root phenolic levels increased by as much as 7%.^59^ The levels of catalase, phenolics, flavonoids, and anthocyanin in soils with elevated Cd concentrations differed greatly between biochar and ascorbic acid. Yet in non-Cd-contaminated soil, phenolics, flavonoids, and anthocyanin were better with biochar and ascorbic acid than with the control [58, 59]. These results are consistent with earlier study; maize pre-treated with indole acetic acid and gibberellic acid had the highest levels of phenolic, followed by maize pre-treated with proline + gibberellic acid and proline + indole acetic acid [60]. The inclusion of various groups, including carboxylic and phenolic groups, can significantly improve the ability of the Fe-BC surface to hold water, conduct cations during cation exchange, and absorb and retain nutrients [61]. Similar to the Cd + Fe-BC treatment, the radish cultivar displayed a decrease (13%) in total shoot phenolic content [62]. Similar studies show that Trichoderma increased buckwheat plants’ levels of gibberellic acid (GA) and indole a-acetic acid (IAA), which in turn increased the production of phenolic chemicals [63]. By dramatically increasing their content in the leaves when compared to the GA_3_ treatment, phenolic compounds appear to be synthesized and built up more easily when biochar is applied. According to research, increasing the dose of biochar above 6% resulted in a decrease in total proline and specifically phenolic content. This was because the excess metals interfered with the function of phenolic compounds in the formation of antioxidants, preventing plants from producing more phenolics [64]. These findings are in line with the research findings. These results are consistent with recent discoveries [65]. By adding Cd, Cu, and Pb to the corn-growing medium, the overall quantity of phenolics at various levels was changed. This shows that greater Cd concentrations may limit the formation of phenolic chemicals in maize plants.

The inhibitory effects of Cd-induced lipid peroxidation and ROS generation, as well as the inhibition of chlorophyll biosynthesis due to Mg ion competition, which damages PS II, could be the cause of reductions in photosynthetic pigments (Chl a, Chl b, and carotenoids) in Cd-stressed plants [66]. The foliar application of Gibberellic acid considerably improved in Chlorophyll a of Desi makai, and the largest fall was observed in Chlorophyll a of Desi makai when 200 m Cd chloride was applied [67]. In comparison to the control, spring corn’s leaf chlorophyll was dramatically decreased. Despite cultivars, *T. asperellum* and biochar applied together improved the leaf chlorophyll an ability to handle Cu toxicity [68]. The chlorophyll a and b contents were positively influenced by rhizobium inoculation and foliar GA_3_ treatment [69]. The effect of toxic metals on chlorophyll b levels was also found to be significant. This aligns with the previous findings, poor Chl biosynthesis and decreased Rubisco protein scavenging activity are other factors contributing to the rate of photosynthesis slowing down under Cd stress [70]. Previous studies have shown a correlation between increased plant biomass, height, and levels of photosynthetic pigment and soil antioxidant effects of ascorbic acid and charcoal [71]. Following our findings, a biochar made from rice husk and maize stalk was applied, together with a kind of bacteria that encourages plant growth, to boost the chlorophyll content of rice plants significantly [72]. Chlorophyll content rose as a result of foliar GA_3_ administration to Rhizobium-inoculated chickpeas [73]. These results show that biochar and GA_3_ may be useful mitigation techniques for the negative impacts of toxic metal Cd toxicity in maize agriculture.

By lowering ROS generation and boosting anti-oxidant activity, BC may reduce oxidative stress [74]. The GA_3_ therapy also resulted in a decrease in root MDA content when compared to the Control group. In plants grown under varying degrees of Cu stress, the activity of SOD, POD, CAT, and APX increased when P and GA_3_ were administered, whereas MDA, H_2_O_2_, EL, and MDA levels in the roots and leaves of *C. capsularis* decreased [75]. The ability of biochar and GA_3_ to increase the resistance of maize plants to toxic metal stress is highlighted by this. According to many studies, higher levels of MDA were found in rice and maize, respectively, due to Cr and Cd toxicity. But by significantly lowering the H_2_O_2_ and MDA levels, charcoal and *T. asperellum* treatment significantly mitigated the impacts of Cu and Ni stress on maize plants [76]. Malondialdehyde (MDA) and H_2_O_2_ levels in a recent study were greater in the control group than they were in the other treatments, contrary to the present study. With GA_3_ (40 mg/L), the best effect was seen, suggesting that it may assist the free radical scavenging mechanism in the leaves of the investigated plants. Cd stress also results in lipid peroxidation and increases MDA formation, both of which disturb the fluidity and integrity of cellular membranes. Our findings concur with that JA and GA_3_ treatment improved the generation of MDA during Cd stress. Combining *T. asperellum* with BC reduced the oxidative stress on the leaves brought on by Cu toxicity. Spring maize demonstrated the largest reduction (86%) in leaf MDA with treatment (BC + *T. asperellum* + Cu) compared to Cu stress [77]. 

## Conclusion

The results of the study reveal the significant impact of cadmium (Cd) stress on various growth parameters and biochemical aspects of maize plants. The initial soil analysis provided baseline data, and the subsequent experiments demonstrated the efficacy of Gibberellic acid (GA_3_) and biochar (BC) in mitigating Cd-induced stress. The decrease in germination percentage under Cd stress was evident, but the application of GA_3_ and BC, both individually and in combination, exhibited positive effects, leading to increased germination rates. Root and shoot lengths were adversely affected by Cd stress, and the treatments involving GA_3_ and BC proved effective in alleviating these negative impacts, with notable improvements observed even under high Cd stress conditions. The protein, phenolic, and chlorophyll contents showed distinct responses to Cd stress, with reductions noted in the presence of Cd. However, GA_3_ and BC treatments demonstrated their potential to ameliorate these reductions and enhance the overall tolerance of maize plants to Cd stress. The findings highlight the promising role of GA_3_ and BC as interventions to enhance Cd tolerance in maize, offering insights into potential strategies for sustainable agriculture in metal-contaminated environments. Further research can explore the underlying mechanisms of these interventions and their broader implications for plant stress physiology and environmental sustainability.

## Data Availability

The author confirms that all data generated or analyzed during this study are included in this published article.

## Declarations

### Study protocol must comply with relevant institutional, national, and international guidelines and legislation

Our experiment follows the relevant institutional, national, and international guidelines and legislation.

## References

[1] Haider FU, Liqun C, Coulter JA, Cheema SA, Wu J, Zhang R, Wenjun M, Farooq M (2021). Toxicity in plants: impacts and remediation strategies. Ecotoxicol Environ Saf.

[2] Abd Elnabi MK, Elkaliny NE, Elyazied MM, Azab SH, Elkhalifa SA, Elmasry S, Mouhamed MS, Shalamesh EM, Alhorieny NA, Abd Elaty AE (2023). Toxicity of Heavy metals and recent advances in their removal: a review. Toxics.

[3] Nawaz A, Nawaz H, Khan K, Haq MU, Khan H, Manan U, Tariq M (2023). Integrated effect of heavy metal-tolerant rhizobacteria and phosphorus on maize growth and phosphorus bioavailability in contaminated soil. J Soil Plant Environ.

[4] Mohamed Salem H, Ali AM (2023). Effect of olive mill wastes on soil physicochemical properties and maize yield under saline soil conditions. J Soil Plant Environ.

[5] Anjum SA (2016). Morpho-physiological growth and yield responses of two contrasting maize cultivars to cadmium exposure. CLEAN-Soil Air Water.

[6] Enyisi IS, Umoh VJ, Whong CMZ, Alabi O, Abdullahi IO (2014). Chemical and nutritional values of maize and maize products obtained from selected markets in Kaduna. J Pharm Allied Sci.

[7] Jiang W, Liu D, Hou W (2001). Hyperaccumulation of cadmium by roots, bulbs and shoots of garlic (*Allium sativum* L). Bioresour Technol.

[8] Tariq M, Iqbal H (2010). Maize in Pakistan-An overview. Agric Nat Resour.

[9] Sandalio LM, Dalurzo HC, Gomez M, Romero-Puertas MC (2001). Del Rio, L. A. Cadmium-induced changes in the growth and oxidative metabolism of pea plants. J Exp Bot.

[10] Sharma P, Dubey RS (2007). Involvement of oxidative stress and role of antioxidative defense system in growing rice seedlings exposed to toxic concentration of aluminum. Plant Cell Rep.

[11] Feng J (2010). Silicon supplementation ameliorated the inhibition of photosynthesis and nitrate metabolism by cadmium (cd) toxicity in *Cucumis sativus* L. Sci Hortic.

[12] Tuna AL, Kaya C, Dikilitas M, Higgs D (2008). The combined effects of gibberellic acid and salinity on some antioxidant enzyme activities, plant growth parameters and nutritional status in maize plants. Environ Exp Bot.

[13] Vahter M (1991). Methods for integrated exposure monitoring of lead and cadmium. Environ Res.

[14] Jaleel CA, Ragupathi G, Rajaram P (2009). Alterations in non-enzymatic antioxidant components of *Catharanthus roseus* exposed to paclobutrazol, gibberellic acid and *Pseudomonas fluorescents*. Plant Omics.

[15] Ghorbanli M, Kaveh SH, Sepehr MF (2000). Effects of cadmium and gibberellin on growth and photosynthesis of *Glycine max*. Photosynthetica.

[16] Shani E (2013). Gibberellins accumulate in the elongating endodermal cells of *Arabidopsis* root. Proce Natl Acad Sci.

[17] Zhang H, Zhang X, Gao G, Ali I, Wu X, Tang M, Chen L, Jiang L, Liang T (2023). Effects of various seed priming on morphological, physiological, and biochemical traits of rice under chilling stress. Front Plant Sci.

[18] Shakir A, Bocianowski J (2023). Enhancing soil fertility of Apple Orchard through Biochar and Fertilizer amendments: a soil aggregation study. J Soil Plant Environ.

[19] Guo G, Lin L, Jin F, Mašek O, Huang Q (2023). Application of heavy metal immobilization in soil by biochar using machine learning. Environ Res.

[20] Shahniza SS, Firdaus MI, Roslan I (2020). Effect of time of application and concentrations of plant growth regulators on growth and yield of sweet corn (*Zea mays* L). Res Crops.

[21] Vwioko ED (2021). Performance of soybean (*Glycine max* L.) variety in salt-treated soil environment following salicylic acid mitigation. J Niger Soc Exp Biol.

[22] Liu Y (2018). Biochar application as a soil amendment for decreasing cadmium availability in soil and accumulation in *Brassica chinensis*. J Soils Sediments.

[23] Yamato M, Okimori Y, Wibowo IF, Anshori S, Ogawa M (2006). Effects of the application of charred bark in *Acacia mangium* on the yield of maize, cowpea, peanut and soil chemical properties in south Sumatra, Indonesia. Soil Sci Plant Nutr.

[24] Rajkovich S (2012). Corn growth and nitrogen nutrition after additions of biochars with varying properties to a temperate soil. Biol Fertil Soils.

[25] Qian TT (2019). Screening of wheat straw biochars for the remediation of soils polluted with zn (II) and cd (II). J Hazard Mater.

[26] Wu C (2018). Adsorption of cadmium on degraded soils amended with maize-stalk-derived biochar. Int J Environl Res Public Health.

[27] Paz-Ferreiro J, Lu H, Fu S, Mendez A, Gasco G (2014). Use of phytoremediation and biochar to remediate heavy metal polluted soils: a review. Solid Earth.

[28] Liu X (2019). Effects of PASP/NTA and TS on the phytoremediation of pyrene-nickel contaminated soil by *Bidens pilosa* L. Chemosphere.

[29] Abedinzadeh M, Etesami H, Alikhani HA, Shafiei S (2020). Combined use of municipal solid waste biochar and bacterial biosorbent synergistically decreases cd (II) and pb (II) concentration in edible tissue of forage maize irrigated with heavy metal–spiked water. Heliyon.

[30] McLean EO (1983). Soil pH and lime requirement. Methods Soil Analysis: Part 2 Chem Microbiol Prop.

[31] Nelson DA, Sommers L, Total Carbon. Organic Carbon, and Organic Matter. Methods Soil Analysis: Part 2 Chem Microbiol Prop. 1983;9:539–79.

[32] Salem LR (2023). Kinetics and adsorption isotherm of strontium on sugarcane biochar and its application in polluted soil. Int J Environ Res.

[33] Rhoades JD, Salinity. Electrical conductivity and total dissolved solids. Methods Soil Analysis: Part 3 Chem Methods. 1996;5:417–35.

[34] Pratt PF, Potassium. Methods Soil Analysis: Part 2 Chem Microbiol Prop. 1965;9:1022–30.

[35] Sultan H, Ahmed N, Mubashir M, Danish S (2020). Chemical production of acidified activated carbon and its infuences on soil fertility comparative to thermo-pyrolyzed biochar. Sci Rep.

[36] Ćwieląg-Piasecka I (2023). Deashed wheat-straw biochar as a potential superabsorbent for pesticides. Materials.

[37] Naz I (2013). Effectiveness of ACC-deaminase containing Pseudomonas strains to induce salinity tolerance in maize under fertilized and unfertilized field conditions. Soil Environ.

[38] Danish S, Zafar-ul-Hye M (2019). Co-application of ACC-deaminase producing PGPR and timber-waste biochar improves pigments formation, growth and yield of wheat under drought stress. Sci rep.

[39] Afrasayab S, Faisal M, Hasnain S (2010). Comparative study of wild and transformed salt tolerant bacterial strains on *Triticum aestivum* growth under salt stress. Braz J Microbiol.

[40] Singleton VL, Rossi JA (1965). Colorimetry of total phenolics with phosphomolybdic–phosphotungstic acid reagents. Am J Enol Vitic.

[41] Arnon DI (1949). Copper enzymes in isolated chloroplasts. Polyphenoloxidase in *Beta vulgaris*. Plant Physiol.

[42] Hasan MK (2015). Melatonin mitigates cadmium phytotoxicity through modulation of phytochelatins biosynthesis, vacuolar sequestration, and antioxidant potential in *Solanum lycopersicum* L. Front Plant Sci.

[43] Velikova V, Yordanov I, Edreva AJP (2000). Oxidative stress and some antioxidant syshoots in acid rain-treated bean plants: protective role of exogenous polyamines. Plant Sci.

[44] d Steel RG, Torrie JH (1986). Principles and procedures of statistics: a Biometrical Approach.

[45] OriginLab, Corporation. OriginPro. OriginLab, Northampton, MA, USA (2021).

[46] Ahmad I, Akhtar MJ, Zahir ZA, Jamil A (2012). Effect of cadmium on seed germination and seedling growth of four wheat (*Triticum aestivum* L.) cultivars. Pak J Bot.

[47] French E, Iyer-Pascuzzi A (2018). S. A role for the gibberellin pathway in biochar-mediated growth promotion. Sci Rep.

[48] Groot SPC, Karssen CM (1987). Gibberellins regulate seed germination in tomato by endosperm weakening: a study with gibberellin-deficient mutants. Planta.

[49] Zhang H, Ullah F, Ahmad R, Shah A, Khan SU, Adnan M (2022). Response of Soil Proteobacteria to Biochar Amendment in sustainable Agriculture- A mini review. J Soil Plant Environ.

[50] Ali I, He L, Ullah S, Quan Z, Wei S, Iqbal A, Ligeng J (2020). Biochar addition coupled with nitrogen fertilization impacts on soil quality, crop productivity, and nitrogen uptake under double-cropping system. Food Energy Secur.

[51] Abdel-Hamid AM, Mohamed HI. The effect of the exogenous gibberellic acid on two salt stressed barley cultivars. Eur Sci J 10 (2014).

[52] Rizvi A, Khan MS (2017). Biotoxic impact of heavy metals on growth, oxidative stress and morphological changes in root structure of wheat (Triticum aestivum L.) and stress alleviation by Pseudomonas aeruginosa strain CPSB1. Chemosphere.

[53] Mohammadzadeh A, Tavakoli M, Motesharezadeh B, Chaichi M (2017). Effects of plant growth-promoting bacteria on the phytoremediation of cadmium-contaminated soil by sunflower. Arch Agron Soil Sci.

[54] Meng H (2009). Cadmium-induced stress on the seed germination and seedling growth of *Brassica napus* L. and its alleviation through exogenous plant growth regulators. Plant Growth Regul.

[55] Amanullah F, Khan WUD (2023). *Trichoderma Asperellum* L. coupled the effects of Biochar to enhance the growth and physiology of contrasting maize cultivars under copper and nickel stresses. Plants.

[56] Akter N, Islam R, Abdul Karim M, Hossain T (2014). Alleviation of drought stress in Maize by Exogenous Application of Gibberellic Acid and Cytokinin. J Crop Sci Biotechnol.

[57] Falkowska M (2011). The effect of gibberellic acid (GA3) on growth, metal biosorption and metabolism of the green algae *Chlorella vulgaris* (Chlorophyceae) Beijerinck exposed to cadmium and lead stress. Pol J Environ Stud.

[58] Mehdizadeh L, Moghaddam M, Lakzian A (2019). Response of summer savory at two different growth stages to biochar amendment under NaCl stress. Arch Agron Soil Sci.

[59] Kovacik J, Backor M (2007). Phenylalanine ammonia-lyase and phenolic compounds in chamomile tolerance to cadmium and copper excess. Water Air Soil Pollut.

[60] Kisa D, Elmastas M, Ozturk L, Kayir O (2016). Responses of the phenolic compounds of *Zea mays* under heavy metal stress. Appl Biol Chem.

[61] Rafique M, Siddiqui MH, Salem MZ (2021). The combined effects of gibberellic acid and rhizobium on growth, yield and nutritional status in chickpea (*Cicer arietinum* L). Agronomy.

[62] Yaseen S (2021). Supplemental effects of biochar and foliar application of ascorbic acid on physio-biochemical attributes of barley (*Hordeum vulgare* L.) under cadmium-contaminated soil. Sustainability.

[63] Dad FP (2020). Influence of iron-enriched biochar on cd sorption, its ionic concentration and redox regulation of radish under cadmium toxicity. Agriculture.

[64] Adejumo SA, Awoyemi V, Togun AO (2020). Exogenous proline and hormone in combination with compost improves growth and tolerance of maize under heavy metal stress. Plants Environ.

[65] Kizito S (2019). Role of nutrient-enriched biochar as a soil amendment during maize growth: exploring practical alternatives to recycle agricultural residuals and to reduce chemical fertilizer demand. Sustainability.

[66] Ancillotti C (2015). Changes in polyphenol and sugar concentrations in wild type and genetically modifed *Nicotiana Langsdorfi* Weinmann in response to water and heat stress. Plant Physiol Biochem.

[67] Rai R, Agrawal M, Agrawal SB. Impact of heavy metals on physiological processes of plants: with special reference to photosynthetic system. Plant Responses Xenobiotics 127–40 (2016).

[68] Naqvi ZB (2020). Effects of gibberellic acid (GA) under cadmium chloride stress on maize (*Zea mays* L). Int J Biol Res.

[69] Khan NA (2016). Ethylene potentiates sulfur-mediated reversal of cadmium inhibited photosynthetic responses in mustard. Front Plant sci.

[70] Noreen S (2021). Foliar fertigation of ascorbic acid and zinc improves growth, antioxidant enzyme activity and harvest index in barley (*Hordeum vulgare* L.) grown under salt stress. Plant Physiol Biochem.

[71] Hafez EM (2019). Synergistic effect of biochar and plant growth promoting rhizobacteria on alleviation of water deficit in rice plants under salt-affected soil. Agronomy.

[72] Mazid M, Khan TA, Mohammad F (2012). Medicinal plants of rural India: a review of use by Indian folks. Indo-Glob Res J Pharm Sci.

[73] Fahad S (2016). A combined application of biochar and phosphorus alleviates heat-induced adversities on physiological, agronomical and quality attributes of rice. Plant Physiol Biochem.

[74] Bashir MA (2021). Biochar mediated-alleviation of chromium stress and growth improvement of different maize cultivars in tannery polluted soils. Int J Environ Res Public Health.

[75] Sun K (2020). Potential of gibberellic acid (Ga3) and uniconazole for enhancing the cd absorption efficiency of maize (*Zea mays* L). Pol J Environ Stud.

[76] Jaiswal D, Pandey A, Agarwal SB. Plant Ecophysiology and Adaptation under Climate Change: Mechanisms and perspectives I, Singapore 515–543 (2020).

[77] Hasan S, Sehar Z, Khan NA (2020). Gibberellic acid and sulfur-mediated reversal of cadmium-inhibited photosynthetic performance in mungbean (*Vigna radiata* L.) involves nitric oxide. J Plant Growth Regul.

